# Thermal Properties of Wood-Plastic Composites with Different Compositions

**DOI:** 10.3390/ma12060881

**Published:** 2019-03-15

**Authors:** Yong Guo, Shiliu Zhu, Yuxia Chen, Dagang Li

**Affiliations:** 1 School of Forestry and Landscape Architecture, Anhui Agricultural University, Hefei 230036, China; zhuslwood@163.com (S.Z.); sheherose@163.com (Y.C.); 2 College of Materials Science and Engineering, Nanjing Forestry University, Nanjing 210037, China

**Keywords:** composites, WPC, wood fiber, rice husk, recycled HDPE, thermal properties

## Abstract

The thermal performance of wood–plastic composites (WPCs) with different fiber, different fiber contents, and different lubricants were investigated in this paper. The results show that the thermal degradation temperature, melting temperature, crystallization temperature, crystallinity, and viscosity of WPCs with wood fiber were slightly higher than those of WPCs with floor sanding powder and rice husk. As the wood fiber content increased, the melting temperature and crystallinity of WPCs decreased while the crystallization temperature, viscosity, and pseudoplasticity increased. When the wood fiber content was increased to 60%, the dimensional stability of WPCs tended to be constant, and a higher wood fiber content was not conducive for processing of WPCs. WPCs had a small coefficient of linear thermal expansion at low temperature and demonstrated a good dimensional stability. The presence of lubricant reduced the viscosity and increased the pseudoplasticity of the WPCs, which is advantageous for the dimensional stability of WPCs at low temperature while making it worse for high temperatures.

## 1. Introduction

As multicomponent composites, there are several factors affecting the service life of wood-plastic composites (WPCs). Among these, one of the crucial factors is the thermal stability of WPCs during their processing and usage. The thermal properties of WPCs are a combination of heat- or temperature-related properties. The research work in the field of the thermal properties of materials primarily includes determination of crystallinity, glass transition temperature, heat deflection temperature, melting behavior (melt flow rate, melting temperature), thermal expansion properties, thermal degradation, and flammability performance.

However, the temperature is not the only factor affecting the thermal stability of WPCs. There are multiple factors that affect the thermal performance of WPCs such as the formulation design [1,2,3,4,5,6], fiber–matrix interface compatibility [7], fiber surface modification [8,9,10,11], and processing aids [12,13,14]; even the heat generated by the cutting forces during the machining process also affects its properties [15]. For instance, thermogravimetric analysis (TGA) results suggested that wood fiber (WF) content is the most crucial factor affecting the thermal stability, initial mass loss, and ash content of composites. Any increase in the wood fiber content leads to an increase in the ash content and less thermally stable composites [1]. In addition, as the wood fiber content and the interface compatibility increases, the thermal expansion coefficient of the WPCs decreases [7,16,17]. Furthermore, both the equilibrium melt temperature and equilibrium torque at steady state increased with increasing WF content but decreased with the addition of lubricant [13]. In the case of other fillers, the incorporation of lignin [18], basalt fiber [3], carbon fiber [19], and vapor-grown carbon nanofibers [20] also enhanced the thermal stability of the polymer matrix. The glass transition temperature, melting point, and degree of crystallinity of the polymers increased with the addition of cellulose nanocrystals or cellulose fibers [21,22,23]. Also, maleic anhydride, maleic anhydride polypropylene copolymer, vinyltrimethoxy silane, and alkalization treated fibers showed better thermal stability as compared to untreated composites [9,24]. 

In actual production units, some lubricants, compatibilizers, or coupling agents are often added to improve the processability and interfacial compatibility of WPCs. The addition of an internal lubricant is effective in reducing the apparent viscosity at high WF loadings [25], and the apparent shear viscosity of the WPC melts decreased gradually with increased lubricant content [26]. Meanwhile, compatibilizers have also been proven to decrease the rheological properties of composites [27]. Studies have shown that the addition of lubricant and maleic anhydride grafted polyethylene (MAPE) resulted in an increase in the measured equilibrium melt temperature and equilibrium torque and improved the extrusion processability of WPCs [13,28]. A combination of HDPE(high-density polyethylene) grade, WF, lubricant, and MAPE contents can provide the benefits of lower shear viscosity while maintaining the mechanical properties and surface smoothness of WPC profiles [26]. In other studies, the introduction of maleic anhydride grafted polypropylene (MAPP) to the system increased the flow behavior of the polymer and decreased the melt viscosity. Meanwhile, the longer the wheat straw fibers, the lower the melt viscosity, while finer wheat straw fibers lead to a high melt viscosity [29]. However, MAPP and particle size have been found to have less impact on the thermal stability, and the melting point had no relationship with WF and MAPP contents and particle size [1].

The material size of WPCs changes under different thermal and temperature conditions. Factors such as the composition of the composites and interaction between the components affect this phenomenon [30]. This has greatly affected the production, engineering design, and service life of products. However, there have been limited comprehensive studies on the thermal performance of WPCs. Therefore, we utilized scientific characterization and testing methods to explore the effects of fiber content, fiber type, and lubricant type on the thermal properties of high-filled WPCs. We are confident that the results of this work will enrich the research of related scientific theories and improve the thermal quality of WPCs.

## 2. Materials and Methods 

### 2.1. Materials

Wood fiber (WF, Italian poplar (*Populus euramevicana* cv. ‘I-214’), in the form of powder) of particle size 40–80 mesh was supplied by Jiangsu Siyang Wood Powder Factory (Siyang, China). Floor sanding powder (FSP, Fir powder (*Cunninghamia lanceolata* (Lamb.) Hook.) of particle size 80–100 mesh was supplied by Shanghai Jiafeng Wood Flour Co., Ltd. (Shanghai, China), while rice husk (RH) with particle size 20–40 mesh was supplied by Anhui Bengbu Rice Husk Processing Factory (Bengbu, China). Recycled high-density polyethylene (Re-HDPE, melt flow index = 1.23 g/10 min at 190 °C, 5 kg) was obtained from Yixing Zhangye Town Huahong Plastic Product Factory (Yixing, China). Maleic anhydride grafted polyethylene (MAPE, CMG9801) was supplied by Shanghai Sunny Technology Co. Ltd. (Shanghai, China) for use as the compatibilizer to improve compatibility between the fiber and Re-HDPE. Stearic acid (HSt) was obtained from Shanghai Yanan Grease Chemical Co., Ltd. (Shanghai, China), while Ethylene Bis Stearamide (EBS) was purchased from Tianxin Nanjing Chemical Co., Ltd. (Nanjing, China). The compound fatty acid salt (CFAS, a mixture of fatty acid ester and fatty acid salt) was purchased from Sinoplas Sunwaychem Chemical Technology Co. Ltd. (Beijing, China). 

### 2.2. Composite Preparation

The WF, FSP, and RH were dried at 105 °C for 12 h to achieve a moisture content of less than 1%. Thereafter, the fibers, Re-HDPE, MAPE, and lubricants were blended and extruded in a co-rotating micro twin-screw extruder (HAAKE Minilab II, Karlsruhe, Germany) as per the desired composition (Table 1, mass fraction) to obtain WPCs. The extrusion process parameters were a feeding speed of 8–10 rpm and a feeding zone temperature of 150–155 °C. The processing temperature was between 170 °C and 180 °C, while the screw speed was in the range of 40–60 rpm. The three lubricants used in the experiments were HSt, MHE (mixture of HSt and EBS, HSt/EBS = 1:1), and CFAS to promote the plasticization of the Re-HDPE matrix and reduce the melt viscosity, leading to an enhanced processing fluidity and surface finish of the product.

### 2.3. Characterization

The thermogravimetric analyses (TGA 209F3, NETZSCH, Selb, Germany) were carried out in a nitrogen protection mode and provided essential information about the thermal properties and degradation behavior of the WPCs. Each composite weighing 10 mg was heated from room temperature to 800 °C at a rate of 10 °C /min. The apparent kinetic parameters were determined by measuring the weight loss behavior during the material decomposition and then employing the Arrhenius Equation (1) to fit this data, where A is the frequency factor, *n* is the reaction order, $E_{a}$ is the apparent kinetic energy of the degradation reaction, *R* is the gas constant (8.314 J/(mol·K)), α is the weight loss rate, and *T* is the absolute temperature. Because the heating rate (β, Equation (2)) is constant, the Arrhenius equation can be written as Equation (3). We integrate Equation (3) to get Equation (4).

(1) $$\frac{d\alpha }{dt}=Aexp\left(-\frac{E}{RT}\right){\left(1-\alpha \right)}^{n}$$

(2) $$\beta =\frac{dT}{dt}$$

(3) dαdt=Aβe−ERT(1−α)n)

(4) dαdt=Aβe−ERT(1−α)n)

(5) $$\ln\left(-\ln\left(1-\alpha \right)\right)=-E_{a}/RT+\ln(RAT_{m}^{2}/\beta E_{a})$$

The thermal decomposition reaction of WPCs is a complicated process. We used the Broido method [31] for the thermodynamic analysis. For a first-order reaction, the Broido equation can be written as Equation (5), where $T_{m}$ is the temperature of maximum reaction velocity. Thus, the activation energy, $E_{a}$, is obtained from the plot of ln(−ln(1−α)) against 1/*T*.

Another 6.0 g sample was tested for thermorheological properties in a co-rotating micromixed rheometer (HAAKE MiniLab II, Karlsruhe, Germany). The temperature was 170–180 °C while the screw speed was 40–60 rpm.

A differential scanning calorimetry (DSC) analysis of the WPCs was performed using a DSC 200F3 differential calorimeter (Netzsch, Selb, Germany) which was fitted with a liquid nitrogen cooling system. The heating and cooling steps were conducted under a N_2_ atmosphere. The samples were heated from –25 °C to 200 °C using a heating rate of 10 °C/min, then held at 200 °C for 5 min (to eliminate the thermal history), then cooled to 25 °C using a cooling rate of 5 °C/min, and finally heated again to 200 °C using a heating rate of 10 °C/min [32,33]. The amount of sample used during the DSC scans was 10 mg. Samples were hermetically sealed in a DSC device prior to being placed into the instrument. The crystallization temperature (T_c_) and melting temperature (T_m_) were determined during crystallization and endothermic melting, respectively, whereas the heat of fusion (ΔH) was calculated from the peak area of the DSC thermogram. The crystallinity (X_c_) of Re-HDPE in the WPCs was calculated from the DSC thermogram according to Equation (6) [34,35], where φ is the amount of filler, ΔH is the heat of fusion of the WPCs (J/g), and ΔH_0_ is the heat of fusion of 100% crystalline HDPE, which was taken to be 293 J/g as per Na et al. [36].

(6) $$X_{c}=100\%\times \frac{\Delta H}{\Delta H_{0}\left(1-\varphi \right)}$$

The thermal expansion performance was tested using a thermomechanical analyzer (TMA 402F1, Netzsch, Selb, Germany) in a tensile loading mode. The samples were cut from the extruded WPCs with dimensions of 15 mm × 4.5 mm × 1 mm [37]. The sample was heated from −40 °C to 150 °C at the heating rate of 5 °C/min, and the process was repeated five times. The onset and endpoint of the glass transition temperature (*T*_g_) and the deformation step height of glass transition (Delta l, the signal difference between the onset and endpoint of the glass transition) were calculated based on the TMA test results [38]. The coefficient of linear thermal expansion (CLTE) was calculated according to Equation (7) [16], where L is the original length of the sample and ΔL/ΔT is the length change rate per degree Celsius.

CLTE = ΔL/(L × ΔT)(7)

## 3. Results and Discussion

### 3.1. Thermogravimetric Analysis (TGA)

**Figure 1:**
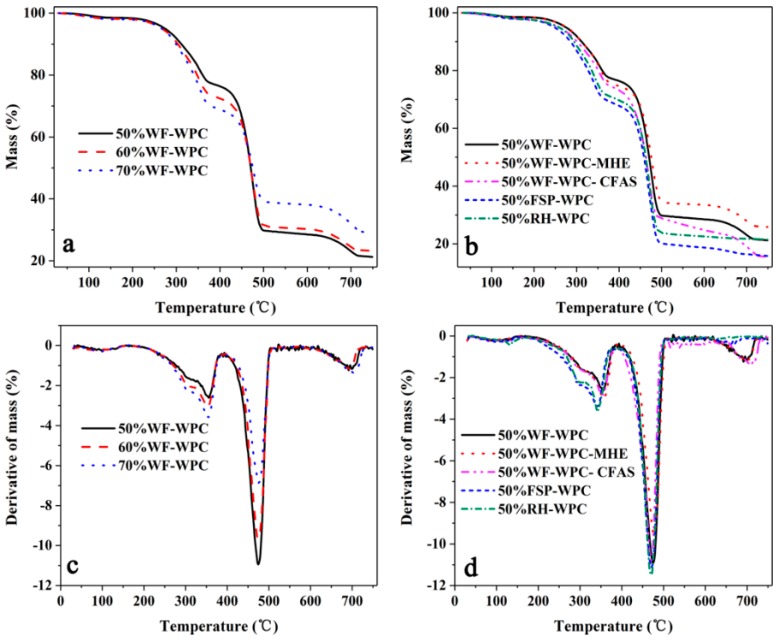
TGA (**a**,**b**) and DTG (**c**,**d**) curves for WPCs.

Figure 1 shows the thermogravimetric analysis (TGA) and digital thermogravimetry (DTG) curves of the WPCs, while the characteristic TGA data are listed in Table 2. The thermal weight loss of different compositions of WPCs was completed in two stages, and the weight loss rate in the second stage was significantly higher than that in the first stage. The decomposition temperature of the first stage was between 290 °C and 370 °C, while that in the second stage was in the range of 440–490 °C. In the first stage, with the increasing WF, the weight loss rate of the WPCs also gradually increased but then gradually decreased in the second stage. This is because the first stage was primarily the decomposition of WF; hence, with the increase of the WF, the weight loss rate of WPCs gradually increased. The second stage was mainly the decomposition of Re-HDPE; therefore, the weight loss rate of WPCs gradually decreased. With increasing WF content, the Re-HDPE coating of WF was gradually reduced. The thermal stability of WF is worse than that of Re-HDPE; therefore, as the WF content increased, the decomposition rate of WPCs in the first stage was gradually accelerated. In the second decomposition stage, the thermal decomposition of Re-HDPE was delayed due to the thermal barrier effect of the carbonized WF. Interestingly, the transformation law of activation energy ($E_{a}$) was basically consistent with the change law of the mass loss rate. However, the value of $E_{a}$ in the second stage was greater than that in the first stage. Moreover, in the second stage, the change of $E_{a}$ has a stronger correlation with the mass loss (or the char residue formation) of the pyrolysis process. These results are primarily due to the reactivity of WF decomposition being greater than that of Re-HDPE decomposition, while substances with high reactivity require less $E_{a}$.

The thermal stability of WPCs with added FSP and RH was not much different, but the initial decomposition temperature in the first stage was clearly lower than that of WPCs with WF while the weight loss rate was apparently higher than that of WPCs with WF. These results indicate that the thermal stability of WF was better than those of FSP and RH. In addition, the results of $E_{a}$ suggest that the reactivity of the three fillers in descending order is FSP, WF, and RH. The decomposition temperature of the WPCs with MHE was apparently higher than those of WPCs with other lubricants and the total weight loss rate was the lowest, suggesting that the addition of MHE was beneficial to improving the thermal stability of WPCs. The influence of MHE on the decomposition speed of the second stage was clearer, indicating that MHE was more conducive to maintaining the thermal stability of Re-HDPE. In addition, the reactivity of MHE was smaller than that of other lubricants at lower temperatures; thus, the $E_{a}$ value of the composites was larger than those of others. However, as the temperature increased, the thermal decomposition of MHE released a large amount of free radicals, which catalyzed the pyrolysis of the composite and increased the reactivity; thus, the $E_{a}$ was reduced in the second stage. For WPCs with CFAS, the initial decomposition temperature in the first stage was close to that of WPCs with HSt, while it was clearly lower in the second stage, indicating that CFAS had an adverse effect on the thermal stability of Re-HDPE. Moreover, the small $E_{a}$ of WPCs with CFAS suggests that the reactivity of CFAS was greater than that of HSt.

### 3.2. Differential Scanning Calorimetry (DSC)

**Figure 2:**
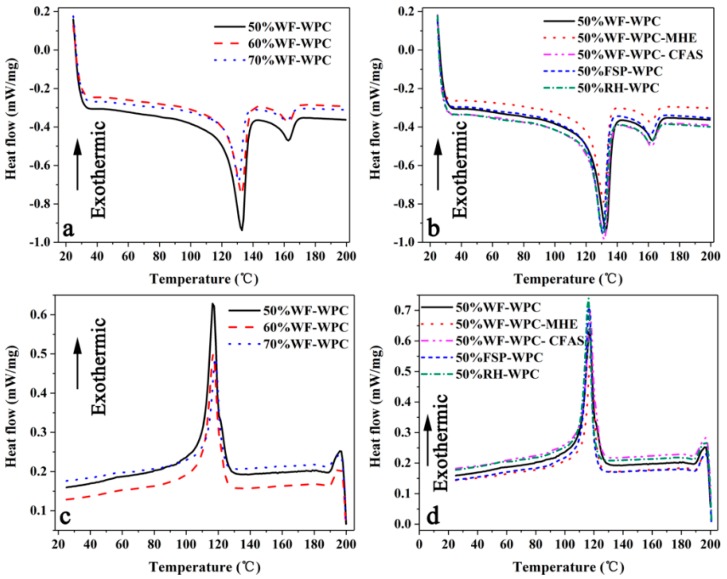
DSC curves for the WPCs (**a**,**b**—melting process; **c**,**d**—crystallization process).

Figure 2 shows the DSC spectra of WPCs of different compositions (a,b—melting process; c,d—crystallization process). The onset of melting temperature (T_om_), the peak melting temperature (T_pm_), the endpoint of melting temperature (T_em_), the melting enthalpy (∆H_m_), the onset of crystallization temperature (T_oc_), the peak crystallization temperature (T_pc_), the endpoint of crystallization temperature (T_ec_), the crystallization enthalpy (∆H_c_), and the crystallinity (X_c_) are given in Table 3 and Table 4. The results show that the WPCs of different compositions had a clear endothermic peak from 25 °C to 200 °C. It can be seen that the endothermic peak was apparently lowered and the melting enthalpy gradually decreased with increased WF content. This is mainly attributed to the dilution effect of the WF within the Re-HDPE matrix [23,25]. On the one hand, due to the reduced relative content of Re-HDPE, the Re-HDPE which produces a heat fusion is reduced. On the other hand, the increased WF limits the thermal movement of the Re-HDPE molecular chain, so the released melting enthalpy is reduced. Different lubricants and fibers had no clear effect on the melting endothermic peak of WPCs. The melting enthalpy of WPCs with WF was higher than that when incorporated with FSP and RH. The melting enthalpy of WPCs incorporated with HSt was higher than those of WPCs using other lubricants, indicating that HSt facilitated the interfacial bonding of WF and Re-HDPE at high temperatures and enhanced the thermal stability.

During the cooling from 200 °C to 25 °C, WPCs with different components had a distinct crystallization peak. The peak crystallization temperature of WPCs increased with increasing WF content but the crystallinity decreased. This is because crystallization is a process in which molecular chains are rearranged. However, an increase in the WF content led to an increase in the viscosity of the Re-HDPE. The addition of WF destroyed the integrity of the Re-HDPE molecular chain, the movement of the molecular chains was blocked, and the regular arrangement area was reduced. The molecular chain is less likely to diffuse into the crystal nucleus and is discharged into the crystal lattice, and the crystal growth rate is lowered; this, in turn, caused the crystallinity to decrease. Moreover, as the WF content increased, the resistance of Re-HDPE chain movement increased, and presenting a regular arrangement became more difficult. Different lubricants and fibers had no clear effect on the peak crystallization temperature of WPCs. However, the crystallinity of WPCs with MHE was apparently lower, and the interface lubrication between the Re-HDPE matrix and WF reduced the formation of a fiber-matrix interfacial crystal layer [39]. In addition, the crystallinity of WPCs with FSP was apparently lower, probably because the fine FSP hindered the polymer crystallization process. The particle size of FSP is smaller, its concentration and distribution range are larger in the composite system, and the hindrance effect on the HDPE chain movement was stronger.

### 3.3. Thermomechanical Analysis

**Figure 3:**
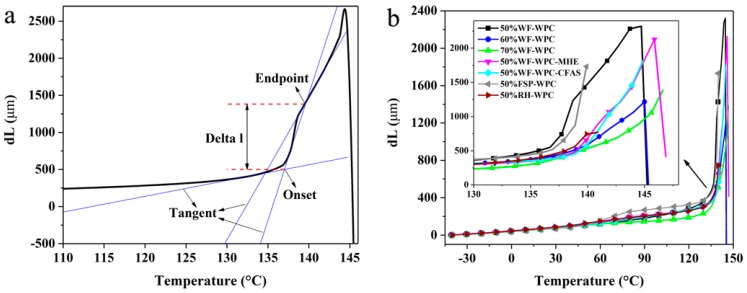
Illustrating of the calculation of the deformation step heights of glass transition (Delta l, **a**) and the curves of dL versus temperature (**b**) acquired through TMA testing.

**Figure 4:**
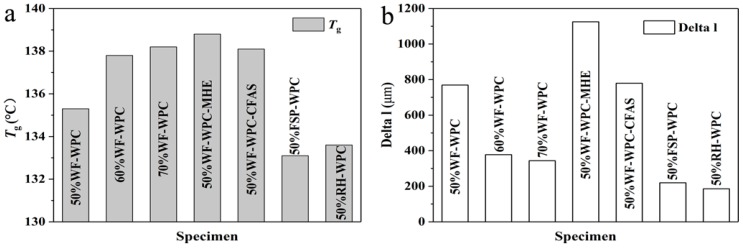
(**a**) The glass transition temperatures (*T_g_*) and (**b**) the deformation step heights of glass transition (Delta l).

The transition of the polymer from the glassy state to rubbery state is a kinetically controlled relaxation process that does not occur at a particular temperature but rather occurs over a wide temperature range. The free volume of the polymer glass state is less than that of the rubbery state, while the coordinated rearrangement in the rubbery state requires additional space. This transformation process has a major impact on the processing and final properties of the materials. Therefore, we analyzed the *T*_g_ and Delta l of WPCs with different compositions, as shown in Figure 3 and Figure 4. The results show that the *T*_g_ of WPCs demonstrated an increasing trend with increasing WF content, while the Delta l was reduced. The deformation produced by glass transition was reduced. These results indicate that increasing the WF content to some extent may reduce the occurrence of defects such as voids in the WPCs. In addition, there were apparent differences in the *T*_g_ values of WPCs with different lubricants: WPCs with HSt had the smallest *T*_g_ while WPCs with MHE had the largest *T*_g_. Therefore, the process temperature should be adjusted at the time of addition of different lubricants. Furthermore, the Delta l of WPCs with MHE was the maximum, which suggests that the glass transition of WPCs with MHE produced larger deformation than that of WPCs with HSt and CFAS and that there is a greater risk of defects in the production process. Also, the appearance of WPCs with HSt and CFAS was clearly better than that of WPCs with MHE. In the case of WPCs with FSP and RH, the *T*_g_ and Delta l values were smaller. These results suggest that the glass transition of WPCs with FSP and RH produced smaller deformation than that of WPCs with WF, and there is a lower risk of defects in the production process. Moreover, the appearance of WPCs with FSP and RH was better.

To further investigate the thermal stability of WPCs in low-temperature, normal-temperature, and high-temperature environments, three temperature ranges of −40 °C to 20 °C, 20 °C to 40 °C, and 40 °C to 80 °C were chosen, and the coefficients of linear thermal expansion (CLTEs) of WPCs in the three temperature zones were calculated (see Table 5). The results show that the CLTE of WPCs displayed an increasing trend with increasing temperature. In the temperature ranges of −40 to 20 °C and 20 to 40 °C, the CLTE of WPCs was relatively low and the difference was small, while in the range of 40 to 80 °C, the CLTE was significantly increased. These results indicate that the WPCs had good dimensional stability under low-temperature and normal-temperature conditions, but this became poor in high-temperature conditions. With increased WF content, the CLTE of WPCs showed a decreasing trend in the three temperature ranges [7,17]. Meanwhile, in the ranges of −40 to 20 °C and 20 to 40 °C, the CLTE values of WPCs with HSt were higher than those of WPCs with MHE and CFAS, while they were relatively lower in 40–80 °C. This phenomenon suggests that the WPCs with MHE and CFAS had better dimensional stability at low and normal temperatures than did WPCs with HSt, but the stability was worse at high temperatures. In addition, in the ranges of −40 to 20 °C and 20 to 40 °C, the CLTE of the WPCs with FSP was the smallest while that of the WPCs with RH was the largest. These results indicate that WPCs with FSP had relatively good dimensional stability under low- and normal-temperature conditions, while the WPCs with RH had relatively inferior dimensional stability. However, in the range of 40–80 °C, the CLTE of the WPCs with FSP was the largest and the CLTE of the WPCs with WF was the smallest, indicating that the dimensional stability of WPCs with WF was better than that of WPCs with FSP at high temperatures. 

**Figure 5:**
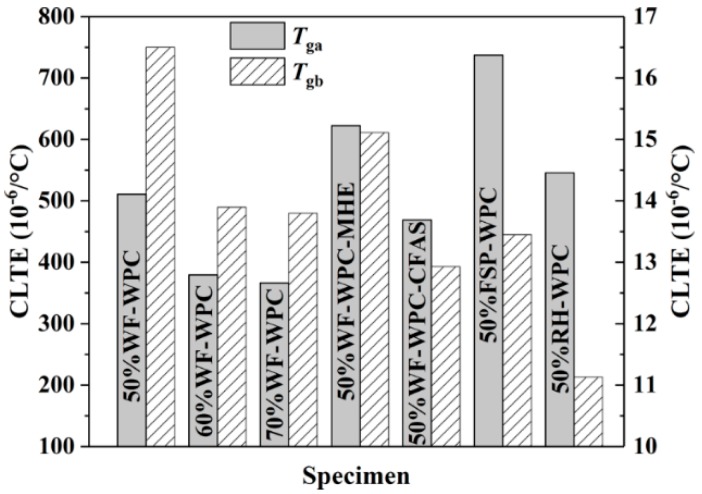
The coefficient of linear thermal expansion (CLTE) of WPCs above and below *T_g_* (*T_ga_*—above *T_g_*; *T_gb_*—below *T_g_*).

As the polymer or composite can maintain its mechanical properties at temperatures below *T*_g_ while being impaired above *T*_g_, when the temperature is lower than *T*_g_, the thermal expansion coefficient of the polymer or composite has more reference value in terms of its mechanical properties. In the temperatures ranges of 20 °C to *T*_g_ and *T*_g_ to 150 °C, the CLTE values of the WPCs showed large differences; the results are shown in Figure 5. In the range of 20 °C to *T*_g_, with increasing WF content, the CLTE of WPCs gradually decreased and tended to be gentle. As the WF content reached 60% or more, the dimensional thermal stability of the WPCs tended to be constant when the temperature was lower than *T*_g_, and the mechanical properties were also relatively easy to maintain. Meanwhile, WPCs with HSt had the highest CLTE while the WPCs with CFAS had the lowest CLTE. This phenomenon suggests that the WPCs with HSt had poor dimensional stability when the temperature was lower than *T*_g_, while the WPCs with CFAS had the best dimensional stability and their mechanical properties could be easily maintained. In addition, WPCs with WF had the largest CLTE while WPCs with RH had the smallest CLTE, indicating that the WPCs with WF had poor dimensional stability when the temperature was lower than *T*_g_, which is not conducive to the maintenance of mechanical properties. Compared to temperatures below *T*_g_, the CLTE of WPCs increased dramatically in the range of *T*_g_ to 150 °C. An increase in CLTE increases the fluidity of the material, making it easier to mold. However, an increase in WF content lowers the CLTE of WPCs. Therefore, an increase in WF content is not conducive for the molding of WPCs. After comparison of different lubricants, we found that the WPCs with MHE had the largest CLTE while the WPCs with CFAS had the smallest CLTE. These results indicate that the addition of MHE helped in enhancing the processing fluidity of WPCs, making them easier to process. The addition of CFAS had little effect on the improvement of process formability. In addition, WPCs with FSP had the largest CLTE, good fluidity during processing, and easy processing.

### 3.4. Thermorheological Analysis

**Figure 6:**
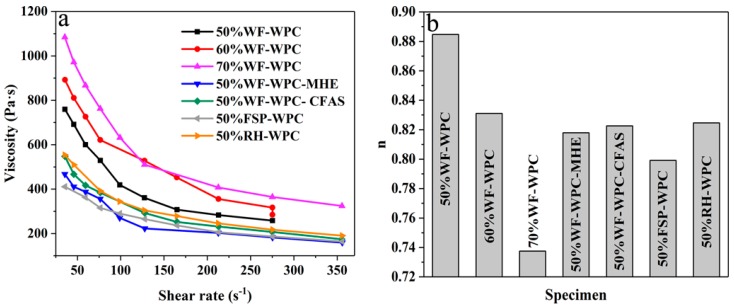
The viscosity and non-Newtonian index (*n*) of WPCs: (**a**) viscosity–shear rate curve; (**b**) *n*.

The viscosity and non-Newtonian index (*n*) of WPCs are shown in Figure 6 (a—viscosity–shear rate curve; b—*n*). The high-filled WPCs were a pseudoplastic fluid [25] when the screw speed was 40–60 rpm. As the shear rate increases, the apparent viscosity decreases. In addition, the apparent viscosity increases rapidly with increasing WF content [25], especially when the shear rate is small. This is primarily because the agglomeration degree of WF increases as the fiber content increases, the resistance to unwinding becomes larger, and the apparent viscosity of WPCs also becomes larger. The lower shear rate cannot effectively unwind the WF, and the severely entangled WF has higher flow resistance to Re-HDPE resin, which causes higher apparent viscosity of the WPCs. As the shear rate increases, the WF is partially disentangled under the action of shearing force. Meanwhile, WF is continuously sheared and oriented in the shear direction. The oriented WF is distributed between the molecular chains of Re-HDPE, which weakens the entanglement between the molecular chains of Re-HDPE; hence, the apparent viscosity decreases with increasing shear rate. These results became more apparent as the WF content was increased.

Lubricants in the WPCs primarily play a role of increasing the spacing between the macromolecules, weakening the intermolecular forces, and reducing the melt flow resistance and apparent viscosity. Figure 6a shows that the lubricants with better lubrication were MHE, CFAS, and HSt in this specified order. The WPCs with MHE had a lower apparent viscosity and could effectively increase the fluidity of the WPC melt. Moreover, the apparent viscosity of WPCs incorporated with WF, RH, and FSP successively decreased. This phenomenon may be related to the size and composition of the fibers. The long fibers with larger size are more prone to agglomeration and entanglement, which hinders the flow of the Re-HDPE resin and causes an increase in the apparent viscosity. Moreover, RH is rich in silicon and silica which can improve the processing fluidity; hence, the apparent viscosity of WPCs with added RH was small. 

According to the setting parameters of Hakke analysis software 3.2.0.1, the relationship between the apparent viscosity of the WPCs and the shear rate is in accordance with the power law formula (Equation (8)), where γ is the shear rate (1/s), η is the apparent viscosity (Pa·s), K is the consistency coefficient (N·s^n^/m^2^), and *n* is the non-Newtonian index.

(8) $$\eta =K\cdot \gamma^{n-1}$$

The value of *n* reflects the shear-thinning ability of the material. The greater the degree to which *n* deviates from 1, the stronger the pseudoplasticity of the material, and the difference between *n* and 1 reflects the strength of the nonlinear properties of the material [40]. Figure 6b shows that the value of *n* decreases with increasing WF content, and the pseudoplasticity of WPCs is enhanced. Moreover, the pseudoplasticity of WPCs incorporated with MHE and FSP was also stronger. The WPCs had a strong dependence on the shearing force and displayed clear visco-shearing properties. At high WF content, the viscosity could be reduced by increasing the shear rate. In turn, this increased the fluidity, improving the processing performance [41] and production efficiency. In addition, the addition of MHE and CFAS could also enhance the processing performance of WPCs.

## 4. Conclusions

We prepared high-filled WPCs by employing WF, FSP, and RH as the fiber reinforcement; Re-HDPE as the matrix; and HSt, MHE, and CFAS as the lubricant. The effects of the WF content, lubricant type, and fiber type on the thermal properties of WPCs were analyzed. The decomposition of WPCs was divided into two stages which were followed by the degradation of fibers and the Re-HDPE matrix. The melting temperature of WPCs decreased as the WF content was increased, while the crystallization temperature increased and the crystallinity decreased. The addition of MHE increased the decomposition temperature of WPCs, slightly decreased the melting temperature, slightly increased the crystallization temperature, and decreased the crystallinity. However, the addition of CFAS reduced the decomposition temperature of Re-HDPE in WPCs. The thermal degradation temperature, melting temperature, crystallization temperature, and crystallinity of WPCs with WF were slightly higher than those of WPCs with FSP and RH.

When the WF content was high, WPCs had a higher *T*_g_, smaller CLTE, and better dimensional stability (especially at 40–80 °C). However, when the WF content was increased to 60%, the dimensional stability of WPCs tended to be constant, and the higher WF content was not conducive to the processing of WPCs. Moreover, as the WF content increased, the viscosity, pseudoplasticity, and shear dependence of WPCs increased. The viscosity of the material could be lowered by increasing the shear rate. In addition, the addition of lubricant was beneficial to the dimensional stability of WPCs at low temperatures while it was not conducive at high temperatures; the addition of MHE and CFAS increased the *T*_g_ of WPCs. Meanwhile, the addition of MHE and CFAS reduced the viscosity of WPCs and increased their pseudoplasticity. The WPCs with FSP were sensitive to the thermal environment and had good dimensional stability at low and normal temperatures but poor dimensional stability at high temperatures. The addition of FSP and RH reduced the viscosity of WPCs.

## Figures and Tables

**Table 1:** The formulation of wood–plastic composites (WPCs) (mass fraction).

Sample	Fiber/%	HDPE/%	MAPE/%	Lubricant/%
50% WF-WPC	50	47	1	2 (HSt)
60% WF-WPC	60	37	1	2 (HSt)
70% WF-WPC	70	27	1	2 (HSt)
50% WF-WPC-MHE	50	47	1	2 (MHE)
50% WF-WPC-CFAS	50	47	1	2 (CFAS)
50% FSP-WPC	50	47	1	2 (HSt)
50% RH-WPC	50	47	1	2 (HSt)

**Table 2:** Characteristic data of the WPCs using thermogravimetric analysis.

Sample	First Decomposition Stage					Second Decomposition Stage				
	T_o_/°C	T_p_/°C	T_e_/°C	Mass loss/%	*E_a_*/kJ/mol	T_o_/°C	T_p_/°C	T_e_/°C	Mass loss/%	*E_a_*/kJ/mol
50%WF-WPC	292.30	355.80	370.20	16.05	14.22	448.80	474.50	489.60	41.33	46.45
60%WF-WPC	292.50	355.70	370.10	19.10	14.84	450.20	474.30	490.40	35.71	40.35
70%WF-WPC	296.00	355.60	369.60	22.00	15.67	451.80	474.10	490.90	24.94	28.33
50%WF-WPC-MHE	299.10	357.10	372.10	17.63	15.16	452.10	477.10	492.20	35.66	39.66
50%WF-WPC-CFAS	293.80	354.40	369.30	16.52	13.27	444.30	471.20	483.70	37.71	41.94
50%FSP-WPC	278.30	341.10	357.70	21.27	13.61	447.50	470.60	486.60	40.31	44.56
50%RH-WPC	286.40	343.50	360.70	20.40	14.57	448.00	471.40	486.00	41.02	45.87

Note: T_o_—temperature at the onset of decomposition; T_p_—the peak decomposition temperature; T_e_—temperature at the endpoint of decomposition; and *E_a_*—activation energy.

**Table 3:** Characteristic data of the WPCs’ melting curves.

Sample	T_om_/°C	T_pm_/°C	T_em_/°C	ΔH_m_/J·g^−1^
50%WF-WPC	122.30	132.90	136.80	48.97
60%WF-WPC	122.70	132.80	135.90	32.67
70%WF-WPC	122.90	130.90	134.80	24.27
50%WF-WPC-MHE	122.30	131.40	135.10	34.16
50%WF-WPC-CFAS	121.80	131.70	135.70	42.98
50%FSP-WPC	121.50	130.40	135.00	42.70
50%RH-WPC	121.50	131.10	135.10	40.64

Note: T_om_—temperature at the onset of melting; T_pm_—the peak melting temperature; T_em_—temperature at the endpoint of melting; and ΔH_m_—the melting enthalpy.

**Table 4:** Characteristic data of the WPCs’ crystal curves.

Sample	T_oc_/°C	T_pc_/°C	T_ec_/°C	ΔH_c_/J·g^−1^	X_c_/%
50%WF-WPC	121.00	116.90	111.00	48.93	33.40
60%WF-WPC	121.40	117.00	112.00	38.26	32.64
70%WF-WPC	122.00	118.10	113.20	27.98	31.83
50%WF-WPC-MHE	122.00	117.90	113.10	38.64	26.37
50%WF-WPC-CFAS	121.50	117.30	112.10	42.90	29.28
50%FSP-WPC	119.80	116.70	112.00	40.86	27.89
50%RH-WPC	119.40	116.10	111.40	46.66	31.85

Note: T_oc_—temperature at the onset of crystallization; T_pc_—the peak crystallization temperature; T_ec_—temperature at the endpoint of crystallization; ΔH_c_—the crystallization enthalpy; and X_c_—the crystallinity.

**Table 5:** The coefficients of linear thermal expansion (CLTEs) of WPCs at different temperatures.

Sample	CLTE (10^−6^/°C)		
	−40~20 °C	20~40 °C	40~80 °C
50%WF-WPC	62.20 ± 2.6	72.00 ± 3.7	101.50 ± 5.2
60%WF-WPC	58.50 ± 3.1	67.50 ± 2.4	82.30 ± 2.9
70%WF-WPC	56.20 ± 2.3	63.90 ± 1.2	71.80 ± 1.9
50%WF-WPC-MHE	57.48 ± 1.8	71.64 ± 2.3	114.86 ± 4.6
50%WF-WPC-CFAS	56.85 ± 2.5	67.32 ± 2.8	106.44 ± 4.9
50%FSP-WPC	56.45 ± 2.2	70.81 ± 2.9	235.78 ± 6.8
50%RH-WPC	65.31 ± 2.8	84.11 ± 3.3	175.43 ± 5.9

## References

[1] Kaboorani A. (2010). Effects of formulation design on thermal properties of wood/thermoplastic composites. Compos. Mater..

[2] Bledzki A., Franciszczak P., Osman Z., Elbadawi M. (2015). Polypropylene biocomposites reinforced with softwood, abaca, jute, and kenaf fibers. Ind. Crops Prod..

[3] Wu Q., Chi K., Wu Y., Lee S. (2014). Mechanical, thermal expansion, and flammability properties of co-extruded wood polymer composites with basalt fiber reinforced shells. Mater. Des..

[4] Jeske H., Schirp A., Cornelius F. (2012). Development of a thermogravimetric analysis (TGA) method for quantitative analysis of wood flour and polypropylene in wood plastic composites (WPC). Thermochim. Acta.

[5] Deng Q., Li J., Yang J., Li D. (2014). Optical and flexible α-chitin nanofibers reinforced poly (vinyl alcohol) (PVA) composite film: Fabrication and property. Compos. Part A Appl. Sci. Manuf..

[6] Xu C., Jian W., Xing C., Zhou H., Zhao Y., Pan H., Xiong X. (2016). Flame retardancy and mechanical properties of thermal plastic composite panels made from T etra P ak waste and high-density polyethylene. Polym. Compos..

[7] Yang H.-S., Wolcott M., Kim H.-S., Kim H.-J. (2005). Thermal properties of lignocellulosic filler-thermoplastic polymer bio-composites. J. Therm. Anal. Calorim..

[8] Nuñez A.J., Kenny J.M., Reboredo M.M., Aranguren M.I., Marcovich N.E. (2002). Thermal and dynamic mechanical characterization of polypropylene-woodflour composites. Polym. Eng. Sci..

[9] Arbelaiz A., Fernandez B., Ramos J., Mondragon I. (2006). Thermal and crystallization studies of short flax fibre reinforced polypropylene matrix composites: Effect of treatments. Thermochim. Acta.

[10] Noura H., Amar B., Hocine D., Rabah Y., Stephane C., Roland E.H., Anne B. (2018). Effect of gamma irradiation aging on mechanical and thermal properties of alfa fiber–reinforced polypropylene composites: Role of alfa fiber surface treatments. J. Thermoplast. Compos. Mater..

[11] Neto J., Lima R., Cavalcanti D., Souza J., Aguiar R., Banea M. (2019). Effect of chemical treatment on the thermal properties of hybrid natural fiber-reinforced composites. J. Appl. Polym. Sci..

[12] Quiles-Carrillo L., Duart S., Montanes N., Torres-Giner S., Balart R. (2018). Enhancement of the mechanical and thermal properties of injection-molded polylactide parts by the addition of acrylated epoxidized soybean oil. Mater. Des..

[13] Feng C., Li Z., Wang Z., Wang B., Wang Z. (2019). Optimizing torque rheometry parameters for assessing the rheological characteristics and extrusion processability of wood plastic composites. J. Thermoplast. Compos. Mater..

[14] Xu C., Xing C., Pan H., Matuana L.M., Zhou H. (2015). Hygrothermal aging properties of wood plastic composites made of recycled high density polypropylene as affected by inorganic pigments. Polym. Eng. Sci..

[15] Guo X., Li R., Cao P., Ekevad M., Cristovao L., Marklund B., Grönlund A. (2015). Effect of average chip thickness and cutting speed on cutting forces and surface roughness during peripheral up milling of wood flour/polyvinyl chloride composite. Wood Res.-Slovak..

[16] Singh S., Mohanty A. (2007). Wood fiber reinforced bacterial bioplastic composites: Fabrication and performance evaluation. Compos. Sci. Technol..

[17] Nakagaito A.N., Yano H. (2008). The effect of fiber content on the mechanical and thermal expansion properties of biocomposites based on microfibrillated cellulose. Cellulose.

[18] Morandim-Giannetti A.A., Agnelli J.A.M., Lanças B.Z., Magnabosco R., Casarin S.A., Bettini S.H. (2012). Lignin as additive in polypropylene/coir composites: Thermal, mechanical and morphological properties. Carbohydr. Polym..

[19] Guo Y., Liu D., Chen Y., Zhang T., Zhu S. (2019). Preparation and properties of carbon-fiber-and pine-cone-fiber-reinforced high-density polyethylene composites. J. Appl. Polym. Sci..

[20] Chipara M., Lozano K., Hernandez A., Chipara M. (2008). TGA analysis of polypropylene–carbon nanofibers composites. Polym. Degrad. Stab..

[21] Siqueira G., Bras J., Follain N., Belbekhouche S., Marais S., Dufresne A. (2013). Thermal and mechanical properties of bio-nanocomposites reinforced by *Luffa cylindrica* cellulose nanocrystals. Carbohydr. Polym..

[22] Siqueira G., Bras J., Dufresne A. (2008). Cellulose whiskers versus microfibrils: Influence of the nature of the nanoparticle and its surface functionalization on the thermal and mechanical properties of nanocomposites. Biomacromolecules.

[23] Amash A., Zugenmaier P. (2000). Morphology and properties of isotropic and oriented samples of cellulose fibre–polypropylene composites. Polymer.

[24] Asim M., Paridah M., Saba N., Jawaid M., Alothman O.Y., Nasir M., Almutairi Z. (2018). Thermal, physical properties and flammability of silane treated kenaf/pineapple leaf fibres phenolic hybrid composites. Compos. Struct..

[25] Li H., Law S., Sain M. (2004). Process rheology and mechanical property correlationship of wood flour-polypropylene composites. J. Reinf. Plast. Compos..

[26] Adhikary K.B., Park C.B., Islam M., Rizvi G.M. (2011). Effects of lubricant content on extrusion processing and mechanical properties of wood flour-high-density polyethylene composites. J. Thermoplast. Compos. Mater..

[27] Sojoudiasli H., Heuzey M.-C., Carreau P.J. (2014). Rheological, morphological and mechanical properties of flax fiber polypropylene composites: Influence of compatibilizers. Cellulose.

[28] Han G., Lei Y., Wu Q., Kojima Y., Suzuki S. (2008). Bamboo–fiber filled high density polyethylene composites: Effect of coupling treatment and nanoclay. J. Polym. Environ..

[29] Pan M., Zhang S., Zhou D. (2010). Melt rheology of wheat straw fiber reinforced polypropylene composites. Acta Mater. Compos. Sin..

[30] Klyosov A.A. (2007). Wood-Plastic Composites.

[31] Broido A. (1969). A simple, sensitive graphical method of treating thermogravimetric analysis data. J. Polym. Sci. Part A-2 Polym. Phys..

[32] Hristov V., Vasileva S. (2003). Dynamic mechanical and thermal properties of modified poly (propylene) wood fiber composites. Macromol. Mater. Eng..

[33] Zhu S., Guo Y., Tu D., Chen Y., Liu S., Li W., Wang L. (2018). Water Absorption, Mechanical, and Crystallization Properties of High-density Polyethylene filled with Corncob Powder. BioResources.

[34] Pandey P., Bajwa S., Bajwa D. (2018). Fiber from DDGS and Corn Grain as Alternative Fillers in Polymer Composites with High Density Polyethylene from Bio-based and Petroleum Sources. J. Polym. Environ..

[35] Wu Y., Tang M., Wang N., Qin J., Chen X., Zhang K. (2018). Preparation and Investigation on Morphology, Thermal Stability and Crystallization Behavior of HDPE/EVA/Organo-Modified Layered Double Hydroxide Nanocomposites. Polym. Compos..

[36] Na B., Zhang Q., Fu Q., Zhang G., Shen K. (2002). Super polyolefin blends achieved via dynamic packing injection molding: The morphology and mechanical properties of HDPE/EVA blends. Polymer.

[37] Glasser W.G., Taib R., Jain R.K., Kander R. (1999). Fiber-reinforced cellulosic thermoplastic composites. J. Appl. Polym. Sci..

[38] Wagner M. (2017). Thermal Analysis in Practice: Fundamental Aspects.

[39] Wolcott M.P., Yin S., Rials T.G. (2000). Using dynamic mechanical spectroscopy to monitor the crystallization of PP/MAPP blends in the presence of wood. Compos. Interfaces.

[40] Lenk R.S. (2012). Polymer Rheology.

[41] Hristov V., Vlachopoulos J. (2007). A study of viscoelasticity and extrudate distortions of wood polymer composites. Rheol. Acta.

